# Transcriptome Analysis of Al-Induced Genes in Buckwheat (*Fagopyrum esculentum* Moench) Root Apex: New Insight into Al Toxicity and Resistance Mechanisms in an Al Accumulating Species

**DOI:** 10.3389/fpls.2017.01141

**Published:** 2017-06-28

**Authors:** Jia Meng Xu, Wei Fan, Jian Feng Jin, He Qiang Lou, Wei Wei Chen, Jian Li Yang, Shao Jian Zheng

**Affiliations:** ^1^State Key Laboratory of Plant Physiology and Biochemistry, College of Life Sciences, Zhejiang UniversityHangzhou, China; ^2^College of Resources and Environment, Yunnan Agricultural UniversityKunming, China; ^3^Institute of Life Sciences, College of Life and Environmental Sciences, Hangzhou Normal UniversityHangzhou, China; ^4^Global Institute for Food Security, University of Saskatchewan, SaskatoonSK, Canada

**Keywords:** aluminum toxicity, buckwheat, cell wall, differential gene expression, organic acids, resistance, transcriptome, transporter

## Abstract

Relying on Al-activated root oxalate secretion, and internal detoxification and accumulation of Al, buckwheat is highly Al resistant. However, the molecular mechanisms responsible for these processes are still poorly understood. It is well-known that root apex is the critical region of Al toxicity that rapidly impairs a series of events, thus, resulting in inhibition of root elongation. Here, we carried out transcriptome analysis of the buckwheat root apex (0–1 cm) with regards to early response (first 6 h) to Al stress (20 μM), which is crucial for identification of both genes and processes involved in Al toxicity and tolerance mechanisms. We obtained 34,469 unigenes with 26,664 unigenes annotated in the NCBI database, and identified 589 up-regulated and 255 down-regulated differentially expressed genes (DEGs) under Al stress. Functional category analysis revealed that biological processes differ between up- and down-regulated genes, although ‘metabolic processes’ were the most affected category in both up- and down-regulated DEGs. Based on the data, it is proposed that Al stress affects a variety of biological processes that collectively contributes to the inhibition of root elongation. We identified 30 transporter genes and 27 transcription factor (TF) genes induced by Al. Gene homology analysis highlighted candidate genes encoding transporters associated with Al uptake, transport, detoxification, and accumulation. We also found that TFs play critical role in transcriptional regulation of Al resistance genes in buckwheat. In addition, gene duplication events are very common in the buckwheat genome, suggesting a possible role for gene duplication in the species’ high Al resistance. Taken together, the transcriptomic analysis of buckwheat root apex shed light on the processes that contribute to the inhibition of root elongation. Furthermore, the comprehensive analysis of both transporter genes and TF genes not only deep our understanding on the responses of buckwheat roots to Al toxicity but provide a good start for functional characterization of genes critical for Al tolerance.

## Introduction

Aluminum (Al) toxicity is one of the major factors limiting crop production on acid soils which comprise about 50% of the world’s arable land (Kochian, 1995). The primary visible symptom of Al toxicity is the inhibition of root elongation which may result from strong binding of Al to and consequent disruption of multiple cellular sites. It has been suggested in the literature that Al toxicity can involve Al interaction with many sites in the root cell, including the cell wall and plasma membrane, binding to and inhibition of enzyme activity and DNA replication, elicitation of toxic reactive oxygen species (ROS), and disruption of signal transduction pathways (Kochian, 1995; Zheng and Yang, 2005; Ma, 2007). Thus, the primary mechanisms of Al toxicity concerning root elongation inhibition remain equivocal. On the other hand, plants have evolved Al-resistance mechanisms to deal with Al toxicity. One of the most important Al-resistance mechanisms relies on the Al-activated secretion of organic acid anions from the root apex, which prevents entry of toxic Al^3+^ ions into growing root tip cells. Depending on plant species, malate, citrate, and oxalate are the major species secreted out of root cells to chelate Al. To date, genes coding for Al-activated malate and Al-activated citrate transporters have been identified from a number of plant species (Liu et al., 2014; Kochian et al., 2015). However, genes involved in Al-induced oxalate secretion have not been isolated in any plant species yet.

There is a great genetic variation for the ability to tolerate Al stress among plant species. Physiologically, Al-resistance mechanisms can be classified as external Al exclusion and internal Al tolerance (Kochian, 1995). Recently, several Al-resistance genes associated with either external exclusion or internal tolerance have been functionally characterized in model plant species, Arabidopsis (*Arabidopsis thaliana*) and rice (*Oryza sativa*) (Delhaize et al., 2012). Arabidopsis is a very Al-sensitive species, whereas rice is very Al resistant. While some Al-resistance genes are conserved between Arabidopsis and rice, others are distinct. For example, mutant approaches have led to the identification of Al-resistance transcription factors (TFs) STOP1 (SENSITIVE TO PROTON RHIZHOTOXICITY1) and ART1 (AL RESISTANCE TRANSCRIPTION FACTOR1) in Arabidopsis and rice, respectively (Iuchi et al., 2007; Yamaji et al., 2009). Both STOP1 and ART1 belong to C2H2-type zinc finger TFs. ART1 regulates the expression of at least 31 genes, and some of them have been demonstrated to be involved in Al tolerance in rice (Yamaji et al., 2009). OsSTAR1 (Sensitive to Aluminum Rhizotoxicity1) and OsSTAR2 form a bacterial-type ABC transporter that alleviates Al toxicity by exporting UDP-glucose to the root cell wall, thereby protecting sensitive cell wall sites from binding Al (Huang et al., 2009). OsNrat1 (Nramp Aluminum Transporter1) and OsALS1 (ALUMINUM SENSITIVE1) operate coordinately to transport Al across plasma membrane (PM) and tonoplast to tolerate Al internally (Xia et al., 2010; Huang et al., 2011). OsFRDL4 (FRD3-Like 4), a root citrate exudation transporter, also alleviates Al externally (Yokosho et al., 2011). OsMGT1 (Magnesium Transporter 1), a Mg transporter, protects root cells from Al toxicity by increasing internal Mg levels, potentially preventing Al from competing with Mg for a number of enzyme binding sites (Chen et al., 2012). OsCDT3 is a small cystein-rich peptide which has binding activity with Al, resulting in the prevention of Al from entering into root cells (Xia et al., 2013). In Arabidopsis, *AtALMT1* (*Aluminum-activated Malate Transporter1*), *AtMATE1* (*Multidrug and Toxin Compound Extrusion1*), *AtALS1, AtALS3*, and *AtSTAR1* have been functionally characterized as Al-resistance genes. Among these, only the expression of *AtALMT1, AtMATE1*, and *AtALS3* are regulated by STOP1 (Liu et al., 2009; Sawaki et al., 2009). Thus, some plant species must have evolved special mechanisms to deal with Al stress, while sharing conserved Al-resistance mechanisms.

Buckwheat is a highly Al-tolerant dicotyledonous plant species (Ma et al., 1997). In addition to exclusion of Al from roots by secretion of oxalate (Zheng et al., 1998), buckwheat accumulates large amounts of Al within leaves without showing obvious toxicity symptoms (Shen et al., 2004). These findings indicate that buckwheat possesses two distinct mechanisms to deal with Al toxicity (root Al exclusion and shoot Al sequestration) in comparison to other plant species such as rice and Arabidopsis. Therefore, understanding the molecular mechanisms of high Al tolerance in buckwheat will help us not only to possibly identify novel Al-tolerance genes, but also better understand the evolution of Al-tolerance mechanisms in plants. However, the genome of buckwheat has not been sequenced and genetic resources are scarce, which hamper the progress of unraveling the molecular basis of Al resistance in buckwheat.

In order to clarify molecular mechanisms of Al toxicity or resistance, several biological techniques such as cDNA-AFLP, suppression subtractive hybridization (SSH), microarray, and RNA-sequencing have been established in a number of plant species. However, only a few of them have been used to functionally characterize *bona fide* Al-resistance genes in contrast with 100s of Al responsive genes (Delhaize et al., 2012). This may have occurred in part because excessively high Al concentrations or long exposure times (or both) was employed in these studies. To address these technical issues in identifying genes directly involved in mechanisms relevant to Al toxicity and tolerance, our group previously employed both low (5 μM) and high (25 μM) Al concentrations in combination with short-term Al exposure (4 h) to construct a SSH library in rice bean (*Vigna umbellata*), and this research provided new insights into Al toxicity and tolerance mechanisms (Fan et al., 2014). Further, several candidate genes were demonstrated to be involved in Al resistance in rice bean. For example, VuSTOP1, a C2H2-type zinc-finger TF was shown to be involved in transcriptional regulation of VuMATE1 (Fan et al., 2015). VuMATE1 has previously been documented as a PM-localized citrate transporter involved in root tip exclusion of Al (Yang et al., 2011c; Liu et al., 2013). Two genes participating in metabolism were also found to be important for Al tolerance in rice bean. VuFDH (*Vigna umbellata* Formate Dehydrogenase), which is specifically involved in formate catabolism, was demonstrated to be important for regulating formate homeostasis under Al stress, because formate accumulation was found to be harmful to root growth (Lou et al., 2016b). VuAAE3 is an oxalyl-CoA synthetase that degrades oxalate to form formate and is also important for Al resistance (Lou et al., 2016a). These results suggest that the control of experimental conditions is critical for identification of novel Al-resistance genes and mechanisms of Al toxicity and tolerance.

Recently, Yokosho et al. (2014) published findings on the transcriptome of buckwheat roots (0–3 cm) and leaves in response to Al stress. It is worth noting that the critical root zone involved in the primary Al stress response is confined to the apical first millimeters of the root (Zheng and Yang, 2005). Thus, it is necessary to further confine the analysis to the root region more specifically associated with Al stress. Zhu et al. (2015) also carried out transcriptome analysis of roots of tartary buckwheat (0–2 and 2–4 cm regions of root) in response to Al stress. However, tartary buckwheat is more Al sensitive in comparison with common buckwheat (Yang et al., 2011b). Therefore, while these studies provided a platform for future research work, our understanding of novel in mechanisms of Al toxicity and resistance employed by buckwheat is still limited. Furthermore, only STOP1/ART1-regulated genes were highlighted, and the comprehensive understanding of Al toxicity and tolerance mechanisms in buckwheat roots remains limited. Therefore, there is a need to identify Al-responsive genes in buckwheat under the appropriate experimental conditions to unravel Al toxicity and resistance mechanisms.

In this present study, we used the RNA-seq technique to characterize Al-responsive genes in buckwheat under short-term (6 h) Al stress conditions that resulted in a moderate Al toxicity, with a focus on the apical root tip (0–1 cm) that is the primary site of Al toxicity. A moderate Al-associated reduction in root elongation was suitable not only for identifying genes associated with resistance mechanisms but also for revealing the initial processes leading to root growth inhibition at the transcriptional level.

## Materials and Methods

### Plant Materials and Growth Conditions

An Al-tolerant buckwheat cultivar (Jiangxi) was used in this study. Seeds were surface sterilized by 5% NaClO for 10 min, and soaked in deionized water overnight after thoroughly rinsed with deionized water. Then, seeds were germinated in the dark at 26°C. Germinated seeds were transferred to a net tray floating on a 5 L of 0.5 mM CaCl_2_ solution (pH 4.5). The solution was renewed daily. For dose-response experiment, 3-day-old seedlings were subjected to 0.5 mM CaCl_2_ solution (pH 4.5) containing 0, 10, 20, 40, or 60 μM AlCl_3_ for 24 h. For time-course experiment, 3-day-old seedlings were subjected to 0.5 mM CaCl_2_ solution (pH 4.5) for 6, 12, or 24 h in the presence or absence of 20 μM AlCl_3_. The growth experiments were carried out in an environmentally controlled growth room with a 14 h/26°C day (light intensity of 300 μmol photons m^-2^ s^-1^) and a 10 h/22°C night regime.

### RNA Isolation and Solexa Sequencing

Total RNA was extracted from frozen root apices using an RNeasy Mini Kit (Qiagen) and digested with RNase free DNAase I (Qiagen). The cDNA library was constructed following the Illumina manufacturer’s instructions. In brief, the polyA+ RNA was purified from total RNA using Oligo(dT) magnetic beads and broken into short fragments using divalent cations at 94°C for 5 min. Using these short fragments as templates, random hexamer-primer was used to synthesize the first-strand cDNA, followed by the synthesis of second-strand cDNA using DNA polymerase I and RNaseH. Short fragments were purified with a QiaQuick PCR Extraction Kit (Qiagen) and ligated to sequencing adapters. The products were amplified by PCR to create a cDNA library. The cDNA library was sequenced using Illumina HiseqTM 2000 system.

### Sequence Assembly and Annotation

The sequencing-received raw image data were transformed by base calling into raw reads. Reads were assembled using Trinity software (Grabherr et al., 2011). The longest assembled sequences were referred to as contigs. Reads were then mapped back to contigs with paired-end reads to detect contigs from the same transcript and the distances between these contigs. The letter N was used to connect each two contigs to represent unknown sequences, and then for Scaffold. Finally, sequences were obtained that lacked N and could not be extended on either end, and were defined as unigenes. The unigene sequences were aligned by BLASTx to protein databases including the NCBI, the Swiss-Prot database, the KEGG database, and COG database (E-value ≤ 10^5^). The sequences of all unigenes were deposited in the NCBI SRA database (accession number: SRA498863).

### Differential Gene Expression Analysis

Gene expression levels were calculated according to the Reads Per kb Million reads (PRKM) method (Mortazavi et al., 2008). This method takes advantage of eliminating the influences of different gene lengths and sequencing discrepancy on the calculation of gene expression level. To identify differential expressed genes (DEGs) between Al-stressed and non-stressed samples, log_2_(fold change) ≥ 1 and RPKM value ≥ 10 were used as the threshold to judge the significance of gene expression difference.

#### Analysis of Gene Ontology Biological Processes

For gene ontology (GO) biological processes analysis, the sequences of DEGs under Al stress were manually subjected to BLASTX search at TAIR web site^1^ and the closest homologs in Arabidopsis were obtained. For each gene homolog, we assigned and classified its GO biological processes according to the TAIR^2^ and AmiGO 2^3^ annotation.

### Quantitative RT-PCR Analysis

Total RNA was extracted using RNeasy Plant Mini Kit (Qiagen). cDNA was synthesized from about 1 μg of total RNA using SuperScript^TM^ reverse transcriptase (Takara, Dalian, China). After appropriate dilution of cDNA samples, 1 μL of cDNA (100 ng μL^-1^) was used as a template for the qRT-PCR in a total volume of 10 μL. Primers for qRT-PCR analysis are listed in Supplementary Table S1, and the PCR amplification conditions were as follows: 94°C for 5 min; 45 cycles of 94°C for 10 s, 55°C for 15 s, and 72°C for 20 s. For each candidate gene, the PCR reactions were carried out in triplicate, and expression data were normalized with expression level of 18S *rRNA*.

## Results

### Effect of Al Stress on Root Growth

For RNA sequencing, preliminary experiments were carried out to choose optimal Al concentrations and exposure durations. As the inhibition of root elongation by Al stress is regarded as the primary visible symptom of Al toxicity, we measured root growth before and after Al stress. In the dose-response experiment, root elongation was progressively inhibited by increasing Al concentrations. Al concentration at 10 μM Al could inhibit root elongation by 10% but this was not statistically significant, whereas 20 μM Al was sufficient to significantly inhibit root elongation by about 20% in comparison with the -Al control after 24 h of exposure (Supplementary Figure S1A). In a time-course experiment, root elongation was inhibited by about 20% after 6 h of exposure to 20 μM Al and this level of inhibition remained constant with prolonged Al exposure time (Supplementary Figure S1B).

### *De novo* Assembly of the Transcripts and Annotation

After cleaning and quality checks of the RNA-seq data, we obtained approximately 53.7 million clean reads with a mean length of 95 bp from the root tips of the control (-Al) library and 66.9 million clean reads with a mean length of 96 bp from the root tips of the Al-treated (+Al) library. Because the genome sequence for buckwheat remains unavailable, a *de novo* assembly method using Trinity software was used (Grabherr et al., 2011). A total of 130,875 transcripts with a mean length of 1,120 bp and 137,601 transcripts with a mean length of 1,081 bp were assembled in the -Al and +Al library, respectively (**Table 1**). Overall, these transcripts represented 34,469 unigenes with transcript length ranging from 201 to 8,394 bp and a mean length of 747 bp (**Table 1**). For annotation, unigene sequences were searched using BLASTx against the NCBI non-redundant protein (Nr) database, with a cut-off E-value of 10^-5^. As a result, 26,664 unigenes (77.36%) were identified with good comparability to known gene sequences (Supplementary Table S2).

**Table 1:** Summary for the buckwheat transcriptome in control (-Al) and Al-treated libraries.

	-Al	+Al
Total number of reads	53,734,590	66,885,294
total base pairs (bp)	5,121,635,232	6,395,201,458
Average read length (bp)	95.31	95.61
Total number of transcripts	130,875	137,601
Mean length of transcripts (bp)	1120 (201–15679)	1081 (201–15679)
Total number of unigenes	34,469	
Mean length of unigenes (bp)	747 (201–8,394)	
Sequence with E-value ≤ 10^-5^	26,664	

### Global Effect of Al Stress on Gene Expression

The expression level of transcripts from the RNA-seq data was calculated by reads per kilobase of exon per million mapped reads (RPKM) method (Mortazavi et al., 2008). For the analysis of Al stress on global gene transcription, the significance of difference in gene expression was judged using log_2_ FC (fold change) ≥ 1 and the absolute value of RPKM ≥ 10 (+Al RPKM ≥ 10 for up-regulated genes and -Al RPKM ≥ 10 for down-regulated genes). On this basis, a total of 844 unigenes were regarded as differentially expressed genes (DEGs) under Al stress, with 589 and 255 unigenes being up- and down-regulated, respectively (Supplementary Table S3).

**FIGURE 1:**
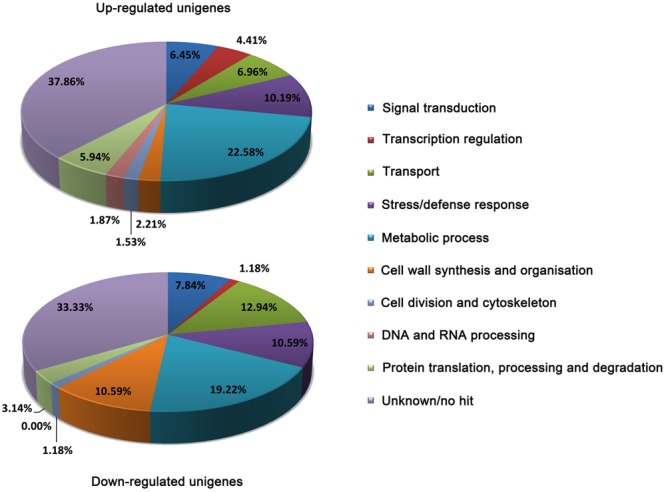
Functional categories of up- and down-regulated DEGs. The classification was performed according to gene ontology (GO) biological process. The percentage of genes in each category was shown.

In order to get a global picture on these DEGs, we performed the BLAST analysis based on Arabidopsis TAIR database^1^ and grouped them into different functional categories according to GO biological process (**Figure 1**). The results showed that 37.86 and 33.33% of the up- and down-regulated DEGs are assigned to unknown function or no hit. In both up- and down-regulated DEGs, genes related to ‘metabolic process’ represent the most affected category, which is consistent with our previous assumption that metabolic changes are critical for plant adaptation to Al stress (Fan et al., 2014). In the group of up-regulated DEGs, genes related to ‘stress/defense response,’ ‘transport,’ and ‘signal transduction’ were the next most abundant categories of up-regulated genes after “metabolic process” genes. Within the down-regulated DEGs, genes involved in ‘transport,’ ‘cell wall synthesis and organization,’ and ‘stress/defense response’ were the next most abundant categories of down-regulated DEGs. Notably, genes related to ‘transcription regulation’ are overwhelming in the up-regulated DEGs (4.41%) against down-regulated DEGs (1.18%), considering the number of up-regulated DEGs are two times more than down-regulated DEGs.

### Validation of Gene Expression by qRT-PCR Analysis

To validate the results of RNA-seq data, 64 DEGs including all up-regulated transporter genes and TF genes were selected for qRT-PCR analysis of transcriptional expression. There was a good correlation (*R*^2^= 0.8198) between RNA-seq data and qRT-PCR results (Supplementary Figure S2), indicating that the RNA-seq data reflected the transcriptional changes caused by Al stress.

### Genes Involved in Metabolism

Metabolism is the foundation of the living cell. We found that genes involved in metabolism are the most represented in both up-regulated and down-regulated DEGs (**Figure 1**). However, genes related to organic acid metabolism were not induced by Al stress, confirming that the transporter rather than metabolism is the rate-limiting factor for Al-induced secretion of organic acids. Interestingly, we found that two genes encoding Acyl Activating Enzyme3 (AAE3) were up-regulated by Al stress. In rice bean, VuAAE3 has been demonstrated to be important for Al resistance by regulating cytoplasmic oxalate homeostasis (Lou et al., 2016a). It appears that oxalate homeostasis regulated by AAE3 is a conserved mechanism for Al resistance in plants (Chen et al., 2017).

### Genes Related to Signal Transduction

An important component of plant response to Al toxicity leading to Al resistance is the triggering and regulation of cellular processes by Al, but the mechanisms by which plants perceive and transduce Al stress signals are poorly understood. Identification of genes related to signal transduction will be helpful for our understanding of plant Al signaling. There are 6.45 and 7.84% of up- and down-regulated DEGs grouped into the ‘signal transduction’ category, respectively (**Figure 1**). Interestingly, the majority of genes were found to be involved in phosphorylation and dephosphorylation of proteins, confirming that reversible protein phosphorylation is an important component of Al resistance (Osawa and Matsumoto, 2001; Kobayashi et al., 2007; Liu et al., 2013). In addition, genes related to Ca^2+^ and hormones signals such as ABA and IAA were also identified to be differentially regulated.

### Genes Encoding Transcription Factors

Because of the importance of TFs in regulating gene expression, TF genes are very abundant in plant genomes and are represented by a large number of families. For example, a total of 72 families of TFs have been identified in Arabidopsis (Mitsuda and Ohme-Takagi, 2009). However, only four types of TFs, C2H2, Abscisic acid, stress and ripening (ASR), CALMODULIN-BINDING TRANSCRIPTION ACTIVATOR (CAMAT), and WRKY genes, have been identified to be involved in Al resistance to date. In this study, we identified 27 genes encoding putative TFs in the up-regulated DEGs category, and these 27 genes belonged to 15 families according to PlnTFDB^4^ (**Table 2**). In addition to known TFs, others may also play a critical role in the induction of expression of Al resistance genes. On the other hand, there were only three TF genes in the down-regulated DEG category (Supplementary Table S3).

**Table 2:** Al up-regulated genes encoding transcription factors.

Gene ID	Log_2_FC	TAIR ID	Annotation	Category^1^
comp17410_c0_seq1	2.61	AT2G36080	Plant-specific B3 DNA-binding domain transcription factor	ABI3VP1
comp3136_c0_seq1	2.45	AT2G36080	Plant-specific B3 DNA-binding domain transcription factor Abscisic acid, stress, and ripening	ABI3VP1
comp30719_c0_seq2	2.05	No hit	Abscisic acid, stress, and ripening	ASR
comp25060_c0_seq1	1.08	AT3G56980	bHLH039	bHLH
comp10926_c0_seq1	1.09	AT1G22490	bHLH094	bHLH
comp21398_c0_seq1	1.14	AT3G56980	bHLH039	bHLH
comp34183_c0_seq1	1.46	AT1G42990	bZIP60	bZIP
comp33784_c0_seq2	1.05	AT5G22890	STOP2	C2H2
comp24031_c0_seq1	1.06	AT5G22890	STOP2	C2H2
comp6278_c0_seq1	1.13	AT4G17570	GATA TRANSCRIPTION FACTOR 26	C2H2-GATA
comp20932_c0_seq1	1.08	AT2G19810	TANDEM ZINC FINGER 2	C3H
comp8554_c0_seq1	1.09	AT2G25900	TANDEM ZINC FINGER PROTEIN 1	C3H
comp57013_c0_seq1	1.55	AT2G22300	CALMODULIN-BINDING TRANSCRIPTION ACTIVATOR 3	CAMTA
comp59172_c0_seq1	1.54	AT2G22300	CALMODULIN-BINDING TRANSCRIPTION ACTIVATOR 3	CAMTA
comp30974_c0_seq1	1.26	AT4G36740	HOMEOBOX PROTEIN 40	HB
comp25409_c0_seq2	2.29	AT5G03720	HEAT SHOCK TRANSCRIPTION FACTOR A3	HSF
comp25147_c0_seq1	1.56	AT4G13980	HEAT SHOCK TRANSCRIPTION FACTOR A5	HSF
comp22296_c0_seq1	2.24	AT5G03720	HEAT SHOCK TRANSCRIPTION FACTOR A3	HSF
comp28871_c0_seq1	1.19	AT3G57230	AGAMOUS-LIKE 16	MADS
comp18677_c0_seq1	1.31	AT5G58900	Homeodomain-like transcriptional regulator	MYB
comp25126_c0_seq1	1.17	AT4G35580	Calmodulin-binding NAC protein	NAC
comp32253_c0_seq1	1.03	AT5G64060	ANAC103	NAC
comp28459_c0_seq1	1.73	AT1G25580	ANAC008/SOG1	NAC
comp33821_c0_seq1	1.26	AT1G01720	ANAC002/ATAF1	NAC
comp29455_c0_seq2	1.18	AT1G01720	ANAC002/ATAF1	NAC
comp15441_c0_seq1	1.08	AT1G01720	ANAC002/ATAF1	NAC
comp24772_c0_seq2	1.40	AT2G38470	WRKY33	WRKY

*^1^PlnTFDB.*

### Genes Encoding Transporters

Transporters are required to participate in the processes of oxalate secretion, and uptake, translocation, compartmentation, and storage of Al. Therefore, we identified all the transporter genes from DEGs up-regulated by Al and found that there were 30 DEGs encoding putative transporters (**Table 3**). According to their potential substrate specificity, these transporters are mainly involved in the transport of phytohormones, organic acids, Al, ions, and sugars. It is noteworthy that gene duplication may be very frequent in the buckwheat genome. For example, there are five and three genes displaying close homology to an ABC transporter and divalent ion transporter, respectively. Also, gene duplication events are present in genes involved in auxin transport, citrate transport, and Mg transport. We observed 33 genes encoding putative transporters in DEGs down-regulated by Al (Supplementary Table S3). In contrast to up-regulated transporters, the down-regulated transporters are mainly involved in water and proton transport. For example, there are seven and six genes belonging to aquaporins and H^+^-ATPase proteins, respectively.

**Table 3:** Al up-regulated genes encoding putative transporters.

Gene ID	Log_2_FC	TAIR ID	Annotation	Substrate^1^
comp74618_c0_seq1	2.46	AT1G15520	ABCG40/ABA transporter	ABA transport
comp21342_c0_seq1	2.32	AT1G15520	ABCG40/ABA transporter	ABA transport
comp59966_c0_seq1	2.16	AT1G15520	ABCG40/ABA transporter	ABA transport
comp79296_c0_seq1	1.86	AT1G15520	ABCG40/ABA transporter	ABA transport
comp5860_c0_seq1	1.17	AT1G15520	ABCG40/ABA transporter	ABA transport
comp24534_c0_seq1	1.58	AT1G02260	Divalent ion transporter	Al hydroxide transport
comp4176_c0_seq1	1.40	AT1G02260	Divalent ion transporter	Al hydroxide transport
comp34167_c0_seq1	1.14	AT1G02260	Divalent ion transporter	Al hydroxide transport
comp34701_c0_seq1	5.24	AT5G03570	IRON-REGULATED PROTEIN 2	Al transport
comp34981_c0_seq5	1.79	AT5G39040	ALS1	Al transport
comp24246_c0_seq1	1.92	AT3G62150	ABCB21	Auxin transport
comp20259_c0_seq1	1.76	AT1G02520	ABCB11	Auxin transport
comp58391_c0_seq1	1.66	AT1G02520	ABCB11	Auxin transport
comp34247_c0_seq1	1.22	AT5G13750	ZINC INDUCED FACILITATOR-LIKE 1	Auxin transport
comp19739_c0_seq1	5.00	AT3G08040	FRD3/MATE	Citrate transport
comp18964_c0_seq1	4.80	AT3G08040	FRD3/MATE	Citrate transport
comp29796_c0_seq1	3.41	AT1G51340	MATE	Citrate transport
comp10304_c0_seq1	2.46	AT5G65380	MATE	Citrate transport
comp20139_c0_seq1	1.10	AT5G52450	MATE	Citrate transport
comp56979_c0_seq1	1.88	AT4G24120	YSL/YSL3/YSL2	Iron transport
comp66023_c0_seq1	1.83	AT5G53550	YSL3	Iron transport
comp74236_c0_seq1	1.03	AT5G41610	CATION/H+ EXCHANGER 18	K^+^ transport
comp9872_c0_seq1	1.72	AT2G27240	ALMT	Malate transport
comp32725_c0_seq2	1.53	AT1G08440	ALMT	Malate transport
comp26205_c0_seq1	4.08	AT5G64560	MGT9	Mg^2+^ transport
comp25699_c0_seq1	3.77	AT5G64560	MGT9	Mg^2+^ transport
comp29702_c0_seq1	5.13	AT2G37330	ALS3/STAR2	UDP-glucose
comp32428_c0_seq1	4.60	AT1G67940	ABCI17/STAR1	UDP-glucose
comp34868_c0_seq3	5.52	AT2G25737	Sulfite exporter TauE/SafE family protein	Unknown
comp54155_c0_seq1	1.45	AT5G59520	ZRT/IRT-LIKE PROTEIN 2	Zn^2+^ transport

*^1^Substrate specificity was assumed according to GO functional analysis.*

### Genes Related to Stress/Defense Response

Genes associated with stress/defense response were identified to be widely affected by Al stress (**Figure 1** and Supplementary Table S3), suggesting that more general stress responses were also activated in buckwheat in response to Al stress. Notably, genes related to oxidative stress were the most overrepresented in both up- and down-regulated DEGs, suggesting that oxidative stress is one of the major factors of Al stress. In addition, genes related to pathogenesis exhibiting different expression patterns also represent a large proportion of genes in this category. However, genes related to osmotic stress were mainly present in the up-regulated DEGs.

### Genes Related to Cell Wall Synthesis and Organization

Genes involved in cell wall synthesis and organization were also identified in this study. While genes encoding cellulose synthases were mostly up-regulated, genes encoding expansins were mostly down-regulated in response to Al (Supplementary Table S3). Expansins are cell wall loosening proteins that have been demonstrated to be important for cell elongation (Che et al., 2016). On the other hand, genes encoding xyloglucan endotransglucosylases/hydrolases (XTHs) and pectin lyase were identified to be regulated differentially by Al. XTHs are xyloglucan-metabolizing enzymes that are believed to be the important agents for controlling cell wall strength and extensibility (Cosgrove, 2005), while pectin lyase degrades pectin polymers directly by β-elimination mechanism that results in the formation of 4, 5-unsaturated oligogalacturonides (Yadav et al., 2009). The differential regulation of XTH and pectin lyase genes suggests that both hemicellulose and pectin metabolism might be important for root elongation (Yang et al., 2008, 2011a).

### Genes Involved in Other Biological Processes

Genes associated with DNA and RNA processing were identified only in the up-regulated DEGs (**Figure 1**). We identified six genes encoding DICER-LIKE 4, that is an RNase III-like enzyme catalyzing processing of *trans-*acting small interfering RNA precursors. Also, there are four genes encoding ABA OVERLY SENSITIVE 4, which is a DNA polymerase essential for DNA replication. In addition, genes responsible for ‘protein translation, processing, and degradation’ were more prevalent in up-regulated DEGs than in down-regulated DEGs.

## Discussion

Buckwheat displays high resistance to Al stress (Ma et al., 1997). Previous physiological studies have demonstrated that both external detoxification and internal tolerance mechanisms are involved in its high Al resistance (Ma et al., 1997, 1998). The internal Al tolerance involves Al transport and sequestration in leaves, indicating that this internal tolerance mechanisms removing Al from the root tip is a relatively complex and coordinated set of processes (Shen et al., 2004). Thus, buckwheat must have evolved a series of coordinately adaptive mechanisms to deal with Al stress. Although recent transcriptome analyses of buckwheat under Al stress have provided transcript sequences and a platform for future functional analysis of genes involved in Al resistance, they focused on transporter genes that may potentially participate in Al tolerance (Yokosho et al., 2014; Zhu et al., 2015). Therefore, the molecular basis of Al toxicity and resistance in this Al-accumulating species still remains far from clear.

It is conceivable that some critical Al toxicity events are initiated at the transcriptional, biochemical, and physiological levels before the onset of root elongation inhibition. Moreover, a series of secondary effects related to Al toxicity occur at or soon after the initiation of root elongation inhibition, especially when higher concentrations or longer treatment times are used. Thus, in order to exclude genes secondarily affected by Al toxicity as much as possible, in this study we carried out transcriptome analysis of buckwheat root apex (0–1 cm) in response to a moderately toxic concentration (20 μM) of Al for a relatively short duration (6 h). In comparison with previous studies, we provided a comprehensive analysis of events possibly involved in Al-induced root growth inhibition. Furthermore, not only transporter genes but also genes encoding TFs were identified from our RNA-seq data, and classified based on their potential functions. In addition, the identified unigenes will be a good complement of previous buckwheat RNA-seq data, providing a platform for further gene functional characterization. Taken together, our present results will not only develop a deeper understanding of Al toxicity and resistance mechanisms in this Al-accumulating species but facilitate the characterization of genes more specifically involved in the primary events of Al stress response.

### Al Toxicity Mechanisms in Buckwheat

Root tip damage and root elongation inhibition are the primary symptoms of Al toxicity in plants. However, the underlying basis of the inhibition is quite complex and remains a matter of debate (Kochian, 1995; Zheng and Yang, 2005; Ma, 2007). It has been suggested that the maintenance of root elongation in the root apex is mainly controlled by three mechanisms: osmotic adjustment, modification of cell wall extension, and accumulation of abscisic acid (ABA) (Sharp et al., 2004; Yamaguchi and Sharp, 2010). Osmotic adjustment is associated with water relations. However, the effects of Al stress on water relations have been little studied. Aquaporins are membrane channels that facilitate the transport of water and small neutral molecules across biomembrane (Maurel et al., 2015). Higher plant aquaporins fall into five subfamilies, i.e., the plasma membrane intrinsic proteins (PIPs), the tonoplast intrinsic proteins (TIPs), the nodulin26-like intrinsic proteins (NIPs), the small basic intrinsic proteins (SIPs), and the uncategorized intrinsic proteins (XIPs). Here, the expression of five PIP genes and two TIP genes was suppressed by Al stress, but none was found in the up-regulated gene group, suggesting that Al stress interferes with the buildup of turgor pressure that provides the driving force for cell elongation.

Among the down-regulated genes, those genes associated with cell wall synthesis and organization were overrepresented (**Figure 1**). Al rapidly reduces cell wall extensibility (Tabuchi and Matsumoto, 2001; Ma et al., 2004), but the underlying mechanisms are still poorly understood. The cell wall extensibility is regulated by its structure as well as the cell wall modifying enzymes such as pectin lyase, expansins, extensins, and XTHs (Cosgrove, 2005). The suppression of genes encoding cell wall modifying enzymes suggests that Al reduces cell wall extension by transcriptional regulation. This is in line with previous reports that the expression suppression of some XTHs might be involved in Al-induced inhibition of root elongation in Arabidopsis (Yang et al., 2011a). On the other hand, some genes belonging to cell wall modifying enzyme categories were also found to be up-regulated. For example, the expression level of three XTH genes and one pectin lyase gene was increased by Al stress (Supplementary Table S3). Also, Al increased the expression of six genes encoding cellulose synthase. As Al may not directly interact with cellulose, the increase in cellulose may reflect the disorder of cell wall metabolism under Al stress, which is critical for cell wall extension.

Although the role of ABA in Al tolerance remains equivocal (Kopittke, 2016), it plays a crucial role for maintenance of root elongation under water deficit (Sharp, 2002; Sharp et al., 2004; Yamaguchi and Sharp, 2010). Recently, Reyna-Llorens et al. (2015) reported that Al could induce the accumulation of ABA in buckwheat root apex, which may serve as a signal to regulate Al resistance by triggering the expression of the Al-tolerance gene, FeALS3. In our study, there were five 5 genes encoding the ABCG40/ABA transporter that were transcriptionally up-regulated by Al stress (**Table 3**). In addition, there are two and four genes involved in ABA signal transduction that were up- and down-regulated, respectively (Supplementary Table S3). These results suggest that Al interferes with ABA homeostasis and signal transduction in buckwheat root apex. However, the role of ABA in buckwheat Al stress response has to be further confirmed.

We also found that a number of genes associated with changes in cytosolic Ca^2+^ were affected by Al stress. For example, a gene encoding autoinhibited Ca^2+^-ATPase 11 were down-regulated by Al stress (Supplementary Table S3). The inhibition of this Ca^2+^-ATPase could result in an increase of cytosolic Ca^2+^, which has generally been suggested to be a cause of Al toxicity (Rengel, 1992; Rengel and Zhang, 2003). In accord with the changes of cytosolic Ca^2+^, there are two and seven genes involved in Ca^2+^ signal transduction that were up- and down-regulated, respectively, by Al stress (Supplementary Table S3).

Out of 33 down-regulated transporter genes, there are six genes encoding H^+^-ATPases (Supplementary Table S3). The PM H^+^-ATPase is an electrogenic pump that exports cellular protons, which not only generates a transmembrane chemical gradient of H^+^, but also establishes an electric gradient energizing secondary transport (Falhof et al., 2016). Thus, it is reasonable to envision that the inhibition of H^+^-ATPase will result in the depolarization of the PM and intracellular acidification. However, direct experimental evidence is still lacking that the suppressed expression of PM H^+^-ATPase is responsible for cytosolic acidification under Al stress. Bose et al. (2010) demonstrated that exposure of Arabidopsis roots to low pH caused membrane depolarization and intracellular acidification possibly through inhibition of PM H^+^-ATPase, whereas a combination of Al stress and low pH had opposing effects. With regards to this topic, it is interesting to note that, the expression of a Cys2His2-type zinc finger TF has been demonstrated to be involved in both Al and low pH tolerance in Arabidopsis (Sawaki et al., 2009) and rice bean (Fan et al., 2015). Also, a gene encoding formate dehydrogenase was found to be induced by both Al stress and low pH in rice bean, and is involved in tolerance to both stresses (Lou et al., 2016b). Besides, some evidence suggests that PM H^+^-ATPase is critical for Al-induced organic acid secretion, though this topic is controversial. Al induces oxalate secretion from buckwheat root apex. However, it is unlikely that the PM H^+^-ATPase activity is directly involved in oxalate secretion, as expression of all PM H^+^-ATPase genes were repressed by Al stress.

Al-induced increases in ROS production have usually been associated with Al toxicity (Yamamoto et al., 2003; Jones et al., 2006). However, the molecular evidence of ROS production under Al stress remains scarce. Here, we found that many genes potentially involved in ROS production were differentially regulated by Al stress at the transcriptional level. In the up-regulated gene group, there are 13 and 17 genes related to oxidation-reduction metabolism and defense against oxidative stress, respectively (Supplementary Table S3). Also, there are 7 and 15 genes related to the two processes respectively in the down-regulated gene grouping (Supplementary Table S3).

**FIGURE 2:**
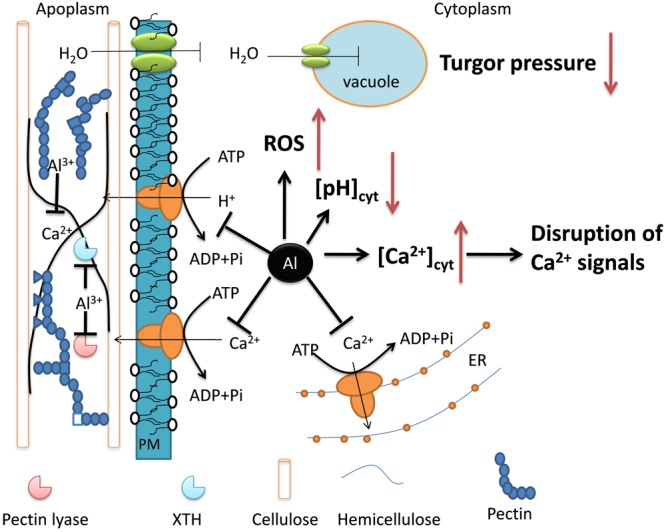
The schematic diagram representing important processes affected by Al stress, which are responsible for Al-induced root growth inhibition. Arrow and bar in black indicate up- and down-regulated changes, respectively, while up- and down-arrow show putatively increased and decreased biochemical and physiological changes, respectively.

On the basis of the present results, we deduce that Al-induced inhibition of root growth is the results of adverse effects of Al stress on a variety of processes. As summarized in **Figure 2**, Al may interfere with cell wall modifying enzymes that reduce cell wall extensibility. Al also inhibits water uptake by inhibiting aquaporins, thus reducing turgor pressure. Additionally, Al can also inhibit PM H^+^-ATPase and Ca^2+^-ATPases, which results in disruption of cellular function and signal transduction. Finally, Al also results in the production of ROS and disruption of ABA metabolism and transport. It appears that Al disrupts a variety of processes that collectively contribute to the inhibition of root elongation.

### Transporters Are Required for High Al Resistance in Buckwheat

**FIGURE 3:**
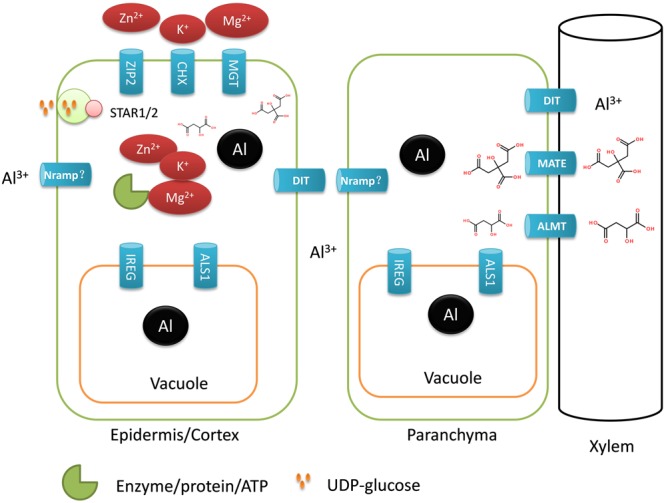
Schematic model for the involvement of putative transporter genes in Al uptake, detoxification, compartmentation, and translocation in buckwheat. ALS1, aluminum sensitive1; DIT, divalent ion transporter; CHX, CATION/H^+^ EXCHANGER; MGT, magnesium transporter; ZIP2; ZINC INDUCED FACILITATOR-LIKE 1; STAR1/2, sensitive to aluminum rhizotoxicity1/2; ALMT, aluminum-activated malate transporter; MATE, multidrug and toxic compound extrusion; IREG, IRON-REGULATED PROTEIN; Nramp, natural resistance-associated macrophage protein; “?” represents remains to be identified.

Buckwheat accumulates a substantial amount of Al in the shoots, suggesting that Al is actively taken up by the roots and translocated from the roots to the shoots; thus, a number of different transporters must be involved. Here, a total of 30 transporter genes were identified to be up-regulated by Al stress (**Table 3**), and some were assumed to be potentially involved in Al uptake, sequestration and distribution (**Figure 3**).

We identified *FeSTAR1* and *FeALS3* encoding an ATP-binding domain and a transmembrane domain of a bacterial type ABC transporter, respectively. In rice, OsSTAR1 and OsSTAR2/ALS3 form a complex to transport UDP-glucose for the modification of cell walls and thereby detoxifying Al apoplastically (Huang et al., 2009). OsMGT1 is reported to be involved in detoxification of Al internally by increasing cytosolic ionic Mg^2+^ and thus helping to prevent displacement of Mg from key intracellular sites by Al (Chen et al., 2012). In the current study, two genes encoding Mg transporter (MGT) proteins were found to be up-regulated by Al stress (**Table 3**). In addition, we found that CHX and ZIP2 genes involved in K^+^ and Zn^2+^ uptake were also upregulated. Cations such as Mg^2+^, Zn^2+^, and K^+^ are important cofactors of enzymes, and Al can also competitively displace these cations and deactivate enzymes that depend on these cations to function (Macdonald and Martin, 1988). The increased expression of transporter genes involved in Mg^2+^, Zn^2+^, and K^+^ uptake suggests the importance of protecting cytosolic enzymes from binding of Al.

With regards to Al uptake, no genes encoding Nramp family transporters were found in the group of up-regulated genes. In rice, an Nrat1 is involved in the uptake of Al into cytosol for external detoxification of Al. In our RNA-seq data, we only found a gene (comp29579_c0_seq1) that displaying 81% identity with Arabidopsis AtNRMAP3 but only 37% identity with rice Nrat1. Furthermore, this contig was only weakly up-regulated by Al stress, suggesting that it is unlikely to be involved in Al uptake. Recently, Negishi et al. (2012) reported the identification of PM-localized Al transporting aquaporin, HmPALT1, in the sepal of the Al accumulator, Hydrangea. However, no aquaporin genes were found to be up-regulated by Al stress in the current study (Supplementary Table S3).

Once taken up, Al needs to be sequestrated into the root and/or leaf vacuole for detoxification. *ALS1* encodes a tonoplast-localized transporter suggested to be involved in sequestration of Al into vacuole (Huang et al., 2011). We identified an Al up-regulated gene showing homology with *ALS1*. Recently, Yokosho et al. (2016) reported that FeIREG1 is another tonoplast-localized transporter that is critical for internalization of Al into vacuole. Here, we also identified FeIREG1 in the Al-induced transporter gene group (**Table 3**). The expression level of *FeIREG1* was dramatically induced by Al stress, suggesting the importance of FeIREG1 in Al tolerance in buckwheat.

Buckwheat accumulates Al in the shoots, suggesting that Al is transported radially through the root to xylem vessels. This can involve both an apoplastic pathway via cell walls and a symplastic pathway through plasmadesmata. The presence of the Casparian band in the endodermis means that at least at the endodermis, a *trans*-PM pathway is required for Al movement to the xylem. Here, we identified three up-regulated genes encoding a putative divalent ion transporter that is a close homolog of the rice silicon efflux transporter Lsi2 (Ma et al., 2007). If this aquaporin protein is involved, chemical form of Al that is transported by this protein is assumed to be neutral. This could be the neutral Al(OH)_3_ species that will be abundant because of the neutral pH of the cytosol, or possibly a neutral form of Al complexed with an organic acid. It is possible that this divalent ion transporter is potentially related to the facilitated diffusion of neutral Al species across a root-cell PM.

We found that the expression of five *MATE* genes was induced by Al stress. MATE protein has demonstrated to be either involved in detoxification of Al or xylem iron translocation from the roots to the shoots, which is controlled by the tissue localization of MATE proteins (Fujii et al., 2012; Liu et al., 2016). Here, the up-regulation of MATE proteins by Al stress suggests that these proteins might be involved in the translocation of Al from the roots to the shoots. Our supposition is based on the following arguments. First, buckwheat roots secrete oxalate instead of citrate to the rhizosphere to chelate Al (Zheng et al., 1998). Second, buckwheat accumulates a substantial amount of Al in the shoots, suggesting that there is a high efficient translocation mechanism for the Al (Shen and Ma, 2001). Finally, it has been demonstrated that Al-citrate complex is the predominant form of Al in the xylem (Ma and Hiradate, 2000).

We also identified two genes encoding ALMT proteins. ALMT proteins have been shown in a number of plant species, including wheat and Arabidopsis, to be PM Al-activated malate efflux transporters involved in Al exclusion form the root tip (Sasaki et al., 2004; Hoekenga et al., 2006). In buckwheat ALMT’s are unlikely to be involved in Al-induced malate secretion into rhizosphere to chelate Al, as little root malate efflux is observed. Thus, the importance of the up-regulation of *ALMTs* has to be investigated. One possible explanation could be that more citrate is required for the xylem translocation of Al when suffering from Al stress, as citrate is also needed for Fe translocation in xylem. Thus, up-regulation of ALMT genes may participate in the release of malate into xylem to coordinate translocation between Al and Fe from the roots to the shoots under Al stress.

### TFs Are Involved in Al-Stress Response in Buckwheat

Although Al rapidly triggers expression changes of 100s of genes, the transcriptional regulation of gene expression remains largely unknown. So far, only a few TF genes have been characterized with respect to Al stress in plants. For example, the C2H2-type zinc finger TF, STOP1/ART1, was the first TFs to be identified that are involved in regulation of Al resistance in plants (Iuchi et al., 2007; Yamaji et al., 2009). In rice, ART1 regulates the expression of at least 31 genes, and some of them have been demonstrated to be involved in Al tolerance (Ma et al., 2014). Also, ASR1 and ASR5 have more recently been shown to complementarily act as a TF independent of ART1 to regulate the expression of Al tolerance genes (Arenhart et al., 2013, 2016). In Arabidopsis, STOP1 regulates not only Al resistance genes such as *AtALMT1* and *AtMATE*, but also genes involved in low pH tolerance (Sawaki et al., 2009). Furthermore, WRKY46 and CALMODULIN-BINDING TRANSCRIPTION ACTIVATOR2 have been demonstrated to act as transcriptional repressor and activator of *AtALMT1* expression, respectively (Ding et al., 2013; Tokizawa et al., 2015). STOP2, a STOP1 homolog, can partially restore Al- and low pH-tolerance by recovering the expression of genes regulated by STOP1 (Kobayashi et al., 2014). Here, we identified not only these known TF homologs such as STOP1, STOP2, CAMTA, WRKY, and ASR, but a number of novel TFs including ABI3VP1, bHLH, C2H2-GATA, C3H, HB, HSF, MADS, MYB and NAC, some of which may be involved in Al resistance and/or Al toxicity (Supplementary Table S2 and **Table 2**). The identification TFs showing close homology to other plant species indicates that Al resistance mechanisms are not surprisingly at least partially evolutionally conserved among plant species. On the other hand, the expression regulation of Al resistance genes also differs among plant species. For instance, in Arabidopsis although the expression of STOP2 was regulated by STOP1, the expression of both STOP1 and STOP2 are not responsive to Al stress. However, in buckwheat the expression of STOP2 was up-regulated by Al, while STOP1 seems to be constitutively expressed (Supplementary Table S2). More interestingly, the expression of the rice bean *VuSTOP1* is responsive to both Al and low pH stress (Fan et al., 2015).

Notably, up-regulated TFs are not always related to Al resistance mechanisms. For example, the SOG1 has been demonstrated as a DNA damage response TF in Arabidopsis (Yoshiyama et al., 2009). However, mutational loss of SOG1 results in increased Al resistance by blockage of Al-dependent DNA damage checkpoint (Sjogren et al., 2015). Here, we found that a gene homologous to SOG1 was up-regulated by Al stress (**Table 2**), indicating that Al stress caused DNA damage rapidly. Thus, the up-regulation of SOG1 is likely the results of Al toxicity in buckwheat.

### Identification of Oxalate Transporter Is Still a Big Challenge

Oxalate secretion has been demonstrated to be involved in Al resistance in a number of plant species, but the transporter responsible for oxalate secretion has not been identified yet. Here, we identified 30 transporter genes, but none of them seems to be a likely candidate for the oxalate transporter (**Table 3**). In similar transcriptome studies with common buckwheat and tartary buckwheat in response to Al stress, there is also no valuable information available for the identification of the oxalate transporter (Yokosho et al., 2014; Zhu et al., 2015). Because there was no lag phase between the onset of Al stress and oxalate secretion (Ma et al., 1997; Zheng et al., 1998, 2005; Yang et al., 2006), Al-induced gene expression may be not required for oxalate secretion. In line with this suggestion, recent reports on wheat ALMT1 and barley HvAACT1 indicate that the expression of genes encoding transporters for malate and citrate secretion is constitutively highly expressed in Al resistant genotypes (Sasaki et al., 2004; Furukawa et al., 2007). Unfortunately, Al-associated secretion of oxalate seems a common mechanism in the genus *Fagopyrum* because tartary buckwheat, wild buckwheat, and common buckwheat all are able to secrete oxalate rapidly from roots under Al stress (Wang et al., 2015). Furthermore, oxalate secretion cannot explain genotypic differences among different cultivars of either common buckwheat (Zheng et al., 2005) or tartary buckwheat (Yang et al., 2011b). Therefore, it is still big challenge to isolate the oxalate transporter gene from plants. An alternative approach could be a genetic approach, based on screening of mutants defective in oxalate secretion, followed by map-based cloning of genes encoding oxalate transporter.

### Gene Duplication Events Contribute to Al Resistance in Buckwheat

We found that the occurrence of gene duplication events is very common in the buckwheat genome. For example, for the known Al resistance TF genes, there are five genes homologous to STOP1/ART1, and two genes homologous to STOP2. With regards to Al up-regulated transporter genes, there are five genes homologous to ABCG40, three genes encoding a divalent ion transporter, two genes encoding FeMGT, and two genes encoding FRD3 (**Table 3**). At present, whether each gene homolog functions independently or redundantly remains unknown. It has been reported that duplication of *HMA4*, a key gene involved in metal translocation and detoxification, is implicated in the evolution of *Arabidopsis halleri* to hyperaccumulate zinc and cadmium (Hanikenne et al., 2008). Also, duplication events are reported to be involved in Al resistance in maize (Maron et al., 2013) and P efficiency in sorghum (Hufnagel et al., 2014). Since common buckwheat is a cross-pollinating species, it is likely that the gene duplication events are frequent and may contribute to high Al tolerance in buckwheat.

In summary, we provided the transcriptome information for the buckwheat root apex in response to Al stress. We highlighted those genes associated with the primary events involved in Al toxicity and Al stress response during the early stages of stress in buckwheat. Our results provide new information concerning biological processes of Al toxicity. The identification of the genes encoding transporters and TFs not only further our understanding of Al resistance mechanisms but provide us a basis for the characterization of novel Al resistance genes.

## Author Contributions

JY and SZ conceived the study. JY and WC designed the experiments. JX, WF, JJ performed the experiments. WF, HL, and JY analyzed the data. JY and WF wrote the manuscript. All authors read and approved the final manuscript.

## Conflict of Interest Statement

The authors declare that the research was conducted in the absence of any commercial or financial relationships that could be construed as a potential conflict of interest.

## Footnotes

1 http://www.arabidopsis.org/Blast/index.jsp

2 http://www.arabidopsis.org/index.jsp

3 http://amigo.geneontology.org/amigo/search/ontology

4 http://plntfdb.bio.uni-potsdam.de/v2.0/index.php?sp_id=ATH
