# Supplementary material for: Transcriptome Analysis of Al-Induced Genes in Buckwheat (Fagopyrum esculentum Moench) Root Apex: New Insight into Al Toxicity and Resistance Mechanisms in an Al Accumulating Species
Source: Front Plant Sci. 2017 Jun 28;8:1141. doi: 10.3389/fpls.2017.01141 (PMC5487443; doi:10.3389/fpls.2017.01141)
Supplement: Supplementary file 4 [file Data_Sheet_1.DOCX]

**Supplemental Figures**


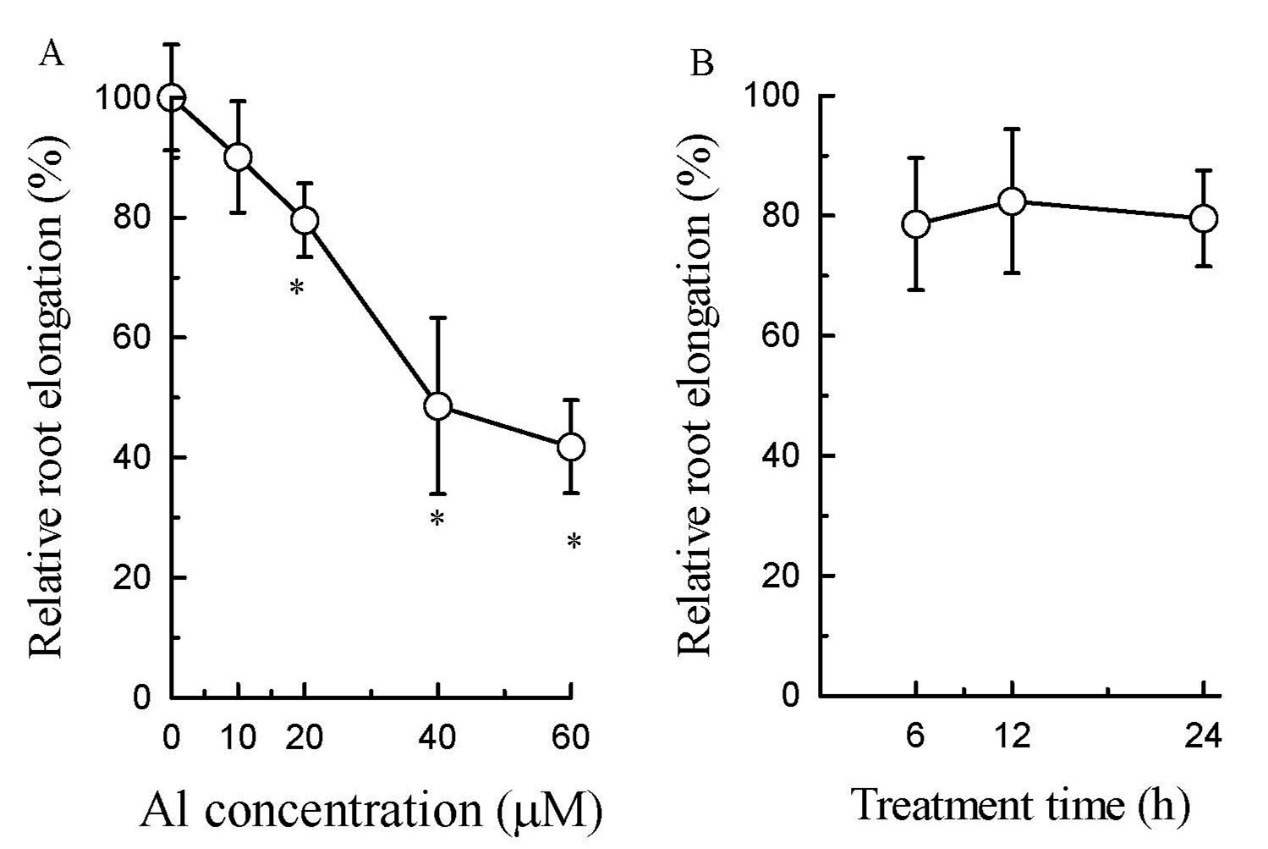


**Supplemental Figure S1.** Effect of Al stress on root elongation inhibition in buckwheat. (A) Three-day-old seedlings were subjected to different dosages of Al for 24 h. root elongation was measured with a ruler before and after treatment. (B) Three-day-old seedlings were subjected to 20 µM Al for different times. The experiment was repeated three times and one set of the results was presented here. Data are expressed as mean ± SD (*n*=16). Asterisks indicate statistically different at *P* < 0.05.


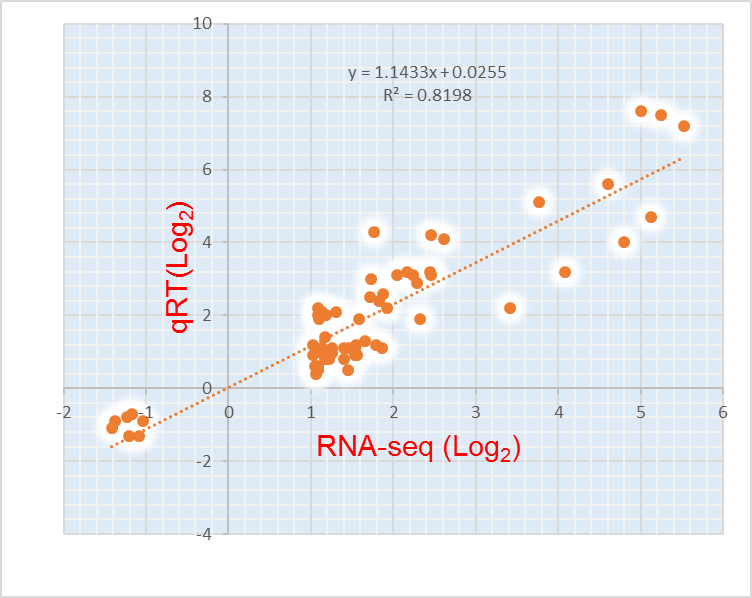


Supplemental Figure S2. Correlation of gene expression levels between RNA-seq data and qRT-PCR analysis. Sixty-four genes including all Al up-regulated genes encoding transporters (30) and transcription factors (27) and seven genes encoding aquaporins were selected and subjected to qRT-PCR analysis using the same RNA as for RNA-seq. 18S rRNA was used as an internal standard. RNA-seq data were plotted against data from qRT-PCR. Both x- and y-axes were shown in log_2_ scale.
